# Supplementary material for: The Impact of the C-Terminal Region on the Interaction of Topoisomerase II Alpha with Mitotic Chromatin
Source: Int J Mol Sci. 2019 Mar 12;20(5):1238. doi: 10.3390/ijms20051238 (PMC6429393; doi:10.3390/ijms20051238)
Supplement: Supplementary file 1 [file ijms-20-01238-s001.pdf]

Suppl Table 1: Human TOP2A expression constructs used/ generated in this study.

Plasmid name	Starting plasmid	Epitope tag	Human TOP2A residues	TOP2A residues mutated	TOP2A residues deleted	Fusion protein	Theor. MW fusion protein (KDa.)	Novel junction sequence(s)
**1. Flag-tagged series**								
pFlag: TOP2A (FT) (Farr et al 2014)	pcDNA3	N-ter 3xFlag	1-1531	WT		-	178	FLAG-(novel)-<br>TOP2A<br>MDYKDHDGDY<br>KDKDIDYKDDD<br>GILQISTVTMEVS<br>PLQP…
pFT-11xKR	pFlag: TOP2A			K→R 1141, 1204, 1228, 1240, 1264, 1267, 1370, 1422, 1442, 1492, 1520			178	
pFT-4xKR	pFlag: TOP2A			K→R 1228 1240 1264 1267			178	
pFT-K1240R	pFlag: TOP2A			K→R 1240			178	
pFT-K662R	pFlag: TOP2A			K→R 662			178	
pFST	pFlag: TOP2A			WT		SUMO2	189	FLAG-(novel)-<br>SUMO2-(novel)-<br>TOP2A<br>MDYKDHDGDY<br>KDHDIDYKDDD<br>GILQIEKPKEGVK<br>ENNDHINLKVA<br>QDGSVVQFKIKR<br>HTPLSKLMKAYC<br>ERQGLSMRQIRF<br>RFDGQPINETDT<br>PALLEMEDEDTI<br>DVFQQQTISTVT<br>MEVSPLQP…
pFTΔ2	pFlag: TOP2A		1-1173::1447-1531	-	1174-1446	-	148	TOP2AΔ<br>DLAT :: LNSGV
pFTΔ3	pFlag: TOP2A		1-1320::1447-1531	-	1321-1446	-	165	TOP2AΔ<br>PRRA :: LNSGV
pFTΔ5	pFlag: TOP2A		1-1211::1447-1531	-	1212-1446	-	152	TOP2AΔ<br>QMAEV :: LNSGV

pFlag-CTR-WT	pFlag:TOP2A		971-1531	WT CTR	1-970	-	67	FLAG-(novel)-TOP2A<br>MDYKDHDGDY<br>KDKDIDYKDDD<br>GLQISKMTEEKL
pFT-CTR-11xKR	pFlag-CTR-WT		971-1531	K→R 1141, 1204, 1228, 1240, 1264, 1267, 1370, 1422, 1442, 1492, 1520	1-970		67	
pFT-CTR-4xKR	pFlag-CTR-WT			K→R 1228 1240 1264 1267			67	
pFT-CTR-3xKR	pFlag-CTR-WT			K→R 1240 1264 1267			67	
pFT-CTR-2xKR	pFlag-CTR-WT			K→R 1228 1240			67	
pFT-CTR-1xKR	pFlag-CTR-WT			K→R 1240			67	
pFT-CTR-T1244A	pFlag-CTR-WT			T→A 1244			67	
pFT-CTR-S1247A	p3xFlag-CTR-WT			S→A 1247			67	
pFT-CTR-TA/SA dm	pFlag-CTR-WT			T→A 1244 S→A 1247			67	
pFT-CTR-TD/SD dm	pFlag-CTR-WT			T→D 1244 S→D 1247			67	

2. GFP-tagged series

pGFP:TOP2A [43]	pEGFP-C1	N-ter EGFP	1-1531	WT			207	GFP-(novel)-TOP2A<br>MVSKGEEL….G<br>MDELYKSGLRSR<br>SRSIVLERFLSPFT<br>TVTMEVSPLQPV<br>….DEDDLF*
pGT-K1240R	pGFP:TOP2A			K→R 1240			207	
pGT-S1213A	pGFP:TOP2A			S→A 1213			207	
pGT-S12147A	pGFP:TOP2A			S→A 1247			207	

pGT-S1213D	pGFP: TOP2A	S→D 1213		207	
pGT-K662R	pGFP: TOP2A	K→R 662		207	
pGT: SUMO2	pGFP: TOP2A	WT	SUMO2	218	GFP-(novel)-TOP2A-(novel)-SUMO2 MVSKGEEL….GMDELYKSGLRSRSRSIVLERFLSPFTTVTMEVSPLQPV….DEDDLFLSDEKPKEGVKTENND….QQQT*
pG:SUMO2:T	pGFP: TOP2A	WT	SUMO2	218	GFP-SUMO2-(novel)-TOP2A MVSKGEEL….GMDELYKADEKPKEGVKTENNDH….DVFQQTISTVTMEVSPLQ….DEDDLF*

In all constructs the selection cassette present confers puromycin-resistance.
